# Pregnancy and epileptic seizures in the emergency department setting: A retrospective analysis

**DOI:** 10.1371/journal.pone.0339402

**Published:** 2026-01-20

**Authors:** Yeliz Simsek, Ayşenur Gur

**Affiliations:** 1 Emergency Department, Adana City Research and Education Hospital, Adana, Turkey; 2 Emergency Department, Etimesgut Sehit Sait Ertürk Hospital, Ankara, Turkey; Foundation IRCCS Carlo Besta Neurological Institute: Fondazione IRCCS Istituto Neurologico Carlo Besta, ITALY

## Abstract

**Background:**

This study evaluated the clinical features, management, and outcomes of pregnant women with generalized tonic-clonic seizures presenting to the emergency department (ED). The aim was to demonstrate how patients’ clinical features contribute to patient management and prognosis.

**Methods:**

In this retrospective study, pregnant women over the age of 18 who presented to the ED with generalized tonic-clonic seizures were included. The patients’ demographic characteristics, clinical findings, treatments administered in the ED, and outcomes were recorded. Descriptive statistics, the chi-square tests or Fisher’s exact tests for categorical variables and the Mann–Whitney U or t-test for continuous variables were used in the statistical analysis.

**Results:**

The study included 48 patients, most of whom were in their third trimester. Thirty-three (69%) patients had a history of epilepsy, and 28 (58.3%) were using antiseizure medications (ASMs). The most commonly used ASM was levetiracetam. Seven (14.6%) patients had suspected eclampsia, and seizure control was achieved in four of them by administering ASMs in addition to magnesium sulfate treatment. Two (4.2%) patients developed status epilepticus (SE). A significant relationship was observed between gestational age and hospitalization (p = 0.005).

**Conclusion:**

The diagnostic complexity of epileptic seizures in pregnancy complicates treatment choices, and ASM use may also be beneficial in managing eclampsia.

## Introduction

Seizures during pregnancy represent a significant cause of maternal and fetal morbidity and mortality. These episodes may result from pre-existing epilepsy, new-onset epilepsy, eclampsia, or a range of secondary etiologies, including cerebrovascular events, postpartum angiopathy, infectious encephalitis, head trauma, intracranial tumors, hepatic failure, metabolic disturbances, drug intoxication or withdrawal syndromes, and autoimmune diseases [1].

Pregnancy is a period of physiological and hormonal changes that can alter the frequency and severity of seizures in women with epilepsy. One-quarter of women with epilepsy experience an increase in seizure frequency [2]. This increased frequency may be associated with various factors, including changes in antiseizure medications (ASM) metabolism, fluctuations in hormone levels, sleep disturbances, and psychological stress. It has been reported that the risk of maternal death in cases of epilepsy during pregnancy is 10 times higher than in the general population [3]. New-onset seizures during pregnancy are rare [4]. The cause of seizures is due to new-onset epileptic seizure or other secondary causes. There is insufficient data on the severity, frequency, or outcomes of these new-onset seizures [1,5].

Status epilepticus (SE) is the most severe form of seizure activity, constituting a life-threatening neurological emergency. SE is a condition resulting from the failure of mechanisms responsible for terminating seizures or from the initiation of mechanisms that lead to abnormally prolonged seizures [6]. During pregnancy, SE can occur in two forms: status epilepticus in women with epilepsy (WWE) and new-onset SE (NORSE) [7]. The reported frequency of status epilepticus in pregnant women with epilepsy ranges from 0% to 1.6% [8,9].

Determining the risk of seizures during pregnancy is crucial for both patient management and clinical decision-making processes. The management of pregnant women experiencing seizures in the emergency department (ED), and the protection of the fetus, is a critical issue that requires a multidisciplinary approach. This study evaluated the clinical characteristics, management approaches, and parameters affecting the prognosis of pregnant women presenting with generalized tonic-clonic seizures in the ED. The aim was to assess the impact of a history of epilepsy on management and prognosis.

## Methods

This was a single-center, retrospective study. The hospital database was screened for pregnant patients who presented to the ED with a chief complaint of seizures between 2021 and 2024. Ethical approval was obtained from the Clinical Research Ethics Committee of Adana Training and Research Hospital (date: August 21, 2025; decision no: 16/670). Data were collected between August 22, 2025, and August 31, 2025. Authors had access to personally identifiable information during the data collection phase; however, all data were fully anonymized before analysis.

Pregnant patients aged ≥18 years who experienced generalized tonic-clonic seizures were included in the study. The definition of generalized tonic–clonic seizures was based on the International League Against Epilepsy (ILAE) seizure classification [10]. Generalized tonic–clonic seizures lasting ≥5 minutes, or recurrent seizures without recovery of consciousness between them, were defined as SE [6,11]. Patients were excluded if they had central nervous system infections, intracranial hemorrhage or infarction, underlying oncological diseases, a history of trauma, or incomplete medical records, to avoid confounding factors and ensure a homogeneous study population.

Patient demographics (age and gestational age), epilepsy history (including prior diagnosis and current ASM use), comorbidities and diagnosis in the ED (classified as prior seizure episode, postictal state, or status epilepticus) were recorded from patient files. Vital signs (arterial blood pressure, pulse rate, respiratory rate, temperature, and oxygen saturation), glasgow coma score (GCS), laboratory parameters at admission, imaging findings (results from emergency obstetric ultrasound, including fetal heart rate, and findings from brain computed tomography (CT) or magnetic resonance (MR) imaging), obstetrics and neurology consultation notes, treatments administered in the ED, and patient outcomes (discharge, hospitalization, delivery, or death) were also collected.

Imaging was performed in patients who did not show clinical improvement, had abnormal neurological findings, a history of immunodeficiency, or were using oral anticoagulants, as well as in those in whom seizures due to secondary causes were suspected based on the physician’s clinical judgment.

Preeclampsia was diagnosed by the presence of pregnancy beyond 20 weeks, proteinuria (≥2+ on a dipstick or >300 mg/24 hours), and arterial hypertension (≥140/90 mmHg). Eclampsia was defined as one or more generalized convulsions and/or coma in a patient with preeclampsia, in the absence of other neurological disorders [12].

Patients were divided into two groups based on whether they had a history of epilepsy. The clinical characteristics and outcomes of the groups were compared.

### Statistical analysis

IBM SPSS Statistics 26.0 software (Statistical Package for the Social Sciences – IBM®) was used for statistical analysis. Data are presented as mean ±standard deviation, minimum-maximum values. Statistical comparisons were performed using the t-test for normally distributed continuous variables and the Mann–Whitney U test for non-normally distributed continuous variables. Categorical variables were analyzed using the chi-square or Fisher’s exact tests as appropriate. A p-value of <0.05 was considered statistically significant.

## Results

A total of 48 patients were included in the study, with a mean age of 27 ± 7.36 years (range:18–45). Patients were distributed across all trimesters: 11 (23%) in the first, 15 (31%) in the second, and 22 (46%) in the third. Fetal heartbeat was confirmed in all patients via emergency ultrasound. All patients presented with complaints of generalized tonic-clonic seizures. Forty patients (83.3%) had their seizures resolved upon presentation, had a GCS score of 15, and demonstrated normal neurological findings. Two patients (4.2%) presented with SE, and six (12.5%) were in the postictal period. None of the patients had fever, and seven (14.6%) patients had a blood pressure above 140/90 mmHg. Laboratory tests did not reveal any metabolic abnormalities that could explain the seizures. All patients underwent neurology and obstetrics consultations, and individualized follow-up and treatment plans were developed accordingly.

Overall 33 (69%) patients had a known history of epilepsy, while 15 (31%) were experiencing their first-ever epileptic seizure. Among patients with a history of epilepsy, the ASMs used were summarized in the Table 1, with the majority levetiracetam (LEV) or lamotrigine (LTG) therapy. No additional comorbidities were noted in these patients. Among the 15 patients without a history of epilepsy, two had comorbidities: one with thalassemia minor and another with hypothyroidism and diabetes. Patients’ age, hematological and biochemical parameters were summarized and compared between patients with and without epilepsy in Table 2. No statistically significant differences were observed.

**Table 1:** Antiseizure medication use among patients with a history of epilepsy.

Antiseizure medication used by patients		Patients with history of epilepsy (n = 33)(%)
Monotherapy	Levetiracetam	13(39)
	Lamotrigine	6(18)
	Carbamazepine	1(3)
	Valproic acid	1(3)
Polytherapy	Levetiracetam +Lamotrigine	4(12)
None		5(15)

**Table 2:** Laboratory results of the patients.

Laboratory Parameters (Normal range)	Patients with history of epilepsy (n = 33)(%)	Patient without a history of epilepsy (n = 15)(%)	*P-value*
	Mean ± Sd	Mean ± Sd	
Glucose (70–100 mg/dL)	108.76 ± 14.35	114.21 ± 29.89	p = 0.41**
Urea (10–50 mg/dL)	16 ± 3.29	15.08 ± 4.88	p = 0.75**
ALT (7–56 U/L)	15.4 ± 5.55	20.8 ± 24.65	p = 0.30**
Sodium (135–145 mmol/L)	139.1 ± 3.4	138 ± 2.3	p = 0.29*
Potassium (3.5–5 mmol/L)	3.91 ± 0.11	3.94 ± 0.11	p = 0.63*
Calcium (8.5–10.2 mg/dL)	8.7 ± 0.63	8.9 ± 0.53	p = 0.17*
Leukocytes (4–11 count/µL)	11 ± 1.2	11 ± 2.2	p = 0.62*
Platelet (150-400x10³/µL)	268.6 ± 112.8	229.3 ± 65.0	p = 0.40**
Hemoglobin (12–16 g/dL)	11.49 ± 4.08	11.05 ± 3.18	p = 0.68**
Lactat (0.5–2.2 mmol/L)	21 ± 21	34 ± 28	p = 0.56**

*t-test was used. **Mann-Whitney U test was used.

ALT: Alanine Aminotransferase, dl: deciliter, g: gram, mg: milligram, L: liter, mL: milliliter, mm, mmol: millimoles, U:unit, µL: microliter.

Cranial MRI was performed in three patients: two with a history of epilepsy who developed SE, and one without a prior diagnosis of epilepsy who experienced a prolonged postictal state. All MRI findings were normal.

Seven patients with suspected eclampsia presented with hypertension, all in the third trimester (Table 3). Among the three patients with a pre-existing diagnosis of epilepsy who developed eclampsia, one was treated with magnesium sulfate and subsequently delivered, while the other two received either LEV alone or a combination of magnesium sulfate and LEV and were discharged after 12 hours of observation in the emergency department. The remaining four patients had no history of epilepsy: two received magnesium sulfate therapy and subsequently delivered, one was treated with a combination of magnesium sulfate and LEV and delivered, and one received LEV alone and experienced a stillbirth.

**Table 3:** Clinic features and management of patient.

Clinical Characteristics of the Patients		Patients with history of epilepsy (n = 33)(%)	Patient without a history of epilepsy (n = 15)(%)	*P-value*
Pregnancy Trimester	First trimester	6(18.2)	5(33.3)	p = 0.511*
	Second trimester	11(33.3)	4(26.7)	
	Third trimester	16(48.5)	6(40)	
Treatment in the Emergency Department	Levetiracetam	27(82)	5(33)	p = 0.006*
	Magnesium	1(3)	2(13)	
	Magnesium+Levetiracetam	2(6)	1(6)	
	None	3(9)	7(46)	
Eclampsia	Yes	3(9)	4(26)	p = 0.101**
	No	30(31)	11(73)	
Emergency department outcomes	Delivery	8(24)	5(33.3)	p = 0.188**
	Discharged	22(66)	7(46.7)	
	Ward admission	1(3)	3(20)	
	Intensive care admission	2(6)	None	

Sd: Standard deviation.

A p-value of <0.05 was considered statistically significant.

*Chi-square test, **Fisher’s exact test.

Two patients (4.2%) experienced SE; both had a history of epilepsy, were in the first trimester, and were on lamotrigine (LTG) monotherapy at presentation. Blood pressure was normal in both cases, and both were monitored in the intensive care unit. One patient received intravenous midazolam and LEV for seizure control, while the other was treated with a combination of midazolam, LEV, and magnesium sulfate.

Treatments administered i in the ED were detailed in Table 3, separately for patients with and without a history of epilepsy. LEV (intravenous 40–60 mg/kg) was administered if there was a missed dose, an increase in seizure frequency, SE, or a second seizure in the ED. Magnesium sulfate (intravenous loading dose of 4–6 gram followed by a continuous maintenance infusion of 1 gram/hour) was given to patients with suspected eclampsia or when the etiology of the seizure was uncertain.

Nineteen patients (39.6%) required hospitalization; among these, 13 (27%) went into labor, with one resulting in a stillbirth. Four patients were admitted for obstetric follow-up, and two patients were admitted to the intensive care unit due to SE. All patients were eventually discharged from the same ward where they were followed. No deaths were reported (Table 3).

A significant association was found between a history of epilepsy and treatment administered in the ED (p = 0.006), with LEV administered to 82% of patients with epilepsy, whereas most patients without epilepsy received no ED treatment. No significant association was observed between a history of epilepsy and hospitalization (p = 0.187; Table 3). No significant relationship was found between ASM use in patients with epilepsy and the need for loading treatment in the ED (p = 0.582) or hospitalization (p = 0.126). There was no significant difference in gestational age between patients with and without a history of epilepsy (p = 0.51; Table 3). However, across all patients, gestational age was significantly associated with hospitalization (p = 0.005), indicating higher admission rates in later trimesters.

## Discussion

Seizures during pregnancy are rare but potentially serious events in emergency settings [13]. They represent one of the most common major neurological disorders during pregnancy and may present either as a breakthrough episode in women with pre-existing epilepsy or as a first-ever epileptic seizure [13,14]. In most women with pre-existing epilepsy, seizure control generally remains stable throughout pregnancy. Approximately 2% of women diagnosed with epilepsy may experience their first epileptic seizure during pregnancy [15]. In this study, we evaluated 48 pregnant women who experienced generalized tonic–clonic seizures. Among them, 33 patients (69%) had a prior history of epilepsy, while 15 patients (31%) experienced their first epileptic seizure. Epileptic seizures were documented across all three trimesters, with the greatest frequency (46%) occurring in the third trimester. Seizures occurring after the 20th week of pregnancy are typically caused by eclampsia, followed by epilepsy, and less frequently, other central nervous system disorders [16]. In our study, seven (14.6%) patients presented with hypertension. Four of these patients received intravenous magnesium sulfate + LEV (and/or benzodiazepine) loading therapy, two patients received only magnesium sulfate, and one patient’s epileptic seizures were controlled with LEV alone. Due to the unavailability of serum drug level measurement at our hospital, seizure treatment was administered based on the patient’s clinical presentation and the clinician’s expert judgment. Diagnostic uncertainty is an expected challenge in pregnant patients presenting with seizures. As demonstrated in our study, this may result in the application of different treatment protocols. The clinical overlap between epileptic seizures and eclampsia can significantly complicate treatment decisions.

Eclampsia is defined generalized convulsions and/or coma in a patient with preeclampsia [12]. Although retrospective studies have indicated an increased risk of preeclampsia in pregnant women with epilepsy, this has not been confirmed in larger studies [17]. While the presence of proteinuria and hypertension in a pregnant patient who has had a seizure may suggest eclampsia, the distinction is not always clear. Notably, hypertension may be absent in 16% of eclampsia patients [18]. In Sibai’s series of 399 women with eclampsia, only 48% of cases had significant proteinuria (≥3+), while 14% had no proteinuria at all [19]. Thornton et al., reported four cases of eclamptic seizures without proteinuria [20]. The literature also reports two case examples of patients diagnosed with preeclampsia and eclampsia who experienced recurrent seizures despite magnesium sulfate therapy but improved with antiepileptic medication. In these cases, hypertension spontaneously resolved after antiepileptic treatment, requiring no further intervention [21]. Furthermore, a case study by Enya S highlighted a situation where the clinical presentation of SE in a pregnant woman with epilepsy was initially mistaken for eclampsia due to elevated blood pressure and proteinuria [22]. Transient proteinuria or labile blood pressure should not be definitively interpreted as eclampsia, especially since eclampsia may necessitate the premature delivery of the fetus [23,24]. The absence of a definitive tool to predict seizures or potential complications significantly increases the challenges in managing this condition [25].

In our center, intravenous antiseizure medication (ASM) loading was generally administered to pregnant women presenting with acute seizures in the emergency department, regardless of previous epilepsy history or serum ASM levels. This approach is preferred to ensure rapid seizure control and prevent maternal–fetal complications. Patients’ serum ASM levels were not measured in the emergency setting. Treatment decisions in this study were largely determined by clinician expertise and individualized clinical judgment. In patients with suspected eclampsia or when the etiology of the seizure was uncertain, magnesium sulfate and/or benzodiazepines were added according to clinical guidelines.

Status epilepticus (SE) is the most severe form of epileptic seizure and is rare, with a reported mortality rate of approximately 28.5% in pregnant women [26,27]. These findings emphasize the critical importance of close monitoring for pregnant women with epilepsy who are at risk of seizures during the prenatal, intrapartum, and postpartum periods. In the cohort of pregnant women with refractory epilepsy reported by Vitturi et al., 8.5% experienced status epilepticus during pregnancy, highlighting the elevated risk in this population [28]. In our study, 2 (4.2%) patients experienced SE, and both were in the first trimester. We observed no patient deaths. This result may be due to our small sample size, especially when compared to a study by Lu YT, which examined seven cases of SE in pregnant women over an eight-year period and reported fatal outcomes [26]. Although SE during pregnancy is rare, there is no single established treatment protocol, and existing data are largely limited to individual case reports or small case series. A review by Rajiv KR, involving 17 pregnant women with pregnancy-related SE over a 15-year period, recommends phenobarbital, phenytoin, and LEV for seizures persisting after magnesium sulfate therapy in eclampsia or after benzodiazepines in non-eclamptic pregnancies [29]. In cases of refractory SE or eclampsia, delivery was recommended irrespective of gestational age [29]. In studies by Roberti et al., benzodiazepines were reported as the first-line treatment for status epilepticus in pregnancy, with levetiracetam and phenytoin considered the most suitable second-line agents [30]. Similarly, Swor et al. found that levetiracetam is the most frequently used anti-seizure medication for benzodiazepine-refractory status epilepticus in pregnant women [31]. In this study, the two patients with SE were discharged after admission to and monitoring in the intensive care unit. Although both patients had normal blood pressure, one patient was treated with a combination of magnesium sulfate, levetiracetam (LEV), and a benzodiazepine, while the other patient received LEV and a benzodiazepine.

Both epilepsy itself and the ASMs used to treat it are associated with maternal and fetal morbidity and mortality [32]. It has been reported that 40% of pregnant women with epilepsy use their medication during pregnancy [17,33]. According to the 2024 update from the EURAP pregnancy registry, 79.0% of women were on monotherapy with a single ASM, of which 36.1% received lamotrigine. Notably, 1.1% of women were not on ASM treatment during the first trimester [34]. In the study by Battino D et al., among pregnancies on monotherapy, worsening seizure control and the need for dose escalation or the addition of another drug were particularly common in LTG-treated pregnancies [9]. Similarly, in the study by Li J et al., the use of a higher number of ASMs during pregnancy was associated with disabling seizures, and lamotrigine use was associated with disabling seizures only when administered as part of polytherapy, not as monotherapy [35]. Voinescu PE and colleagues found that women with focal epilepsy and those receiving polytherapy during pregnancy had a higher likelihood of seizure worsening during pregnancy [36]. Conversely, a study involving 57 pregnant women receiving monotherapy observed better clinical outcomes during pregnancy compared to the pre-pregnancy period, starting from the first trimester. Seizure freedom during pregnancy was found to be higher in those using newer ASMs such as LTG and LEV compared to those using older agents like valproic acid and carbamazepine [37,38]. Although LTG is generally considered a safe option during pregnancy, these findings indicate that its serum levels can fluctuate during gestation, necessitating close monitoring. In our study, the patients who presented with status epilepticus had epilepsy and were receiving lamotrigine monotherapy. The patients with SE in the study were in their first trimester and may have stopped taking their medication regularly due to anxiety about drug use during pregnancy or labile serum drug levels during pregnancy may have contributed to this clinical presentation. According to our study, twenty-four (50%) patients were on a single ASM, and 4 (8.4%) were on multiple ASMs. The most commonly used drugs were LEV and LTG. The fact that the majority of patients were using newer-generation ASMs suggests that these medications are more frequently preferred during pregnancy. Despite the limited sample size, our findings contribute to underscore the need for larger, prospective studies on seizure management during pregnancy.

## Limitations

This study has several limitations, primarily due to its small sample size and retrospective design. Information on the previous epileptic attacks of patients diagnosed with epilepsy was not available. Furthermore, seizure types that were not part of the study’s inclusion criteria, as well as seizures occurring during the postpartum period, were not assessed. Data regarding maternal and fetal follow-up after hospital discharge were unavailable. No electroencephalography examinations could be performed in the ED.

## Conclusion

Seizures were most frequently observed during the third trimester, with no significant difference between pregnant women with and without a pre-existing diagnosis of epilepsy. Our findings suggest that newer-generation ASMs, particularly levetiracetam and lamotrigine, are commonly preferred during pregnancy. The occurrence of status epilepticus in patients receiving lamotrigine monotherapy highlights the need for close clinical and therapeutic monitoring, especially during the first trimester. According to our results, neither a prior epilepsy diagnosis nor ASM use significantly influenced outcomes or treatment requirements in the emergency department. The diagnostic overlap between epileptic seizures and eclampsia remains a major clinical challenge and requires careful evaluation.

## References

[1] Hart LA, Sibai BM. Seizures in pregnancy: epilepsy, eclampsia, and stroke. Semin Perinatol. 2013;37(4):207–24. doi: 10.1053/j.semperi.2013.04.001 23916020

[2] Pennell PB. Pregnancy, epilepsy, and women’s issues. Continuum (Minneap Minn). 2013;19(3 Epilepsy):697–714. doi: 10.1212/01.CON.0000431383.14061.e6 23739105 PMC10563998

[3] Edey S, Moran N, Nashef L. SUDEP and epilepsy-related mortality in pregnancy. Epilepsia. 2014;55(7):e72-4. doi: 10.1111/epi.12621 24754364

[4] Beach RL, Kaplan PW. Seizures in pregnancy: diagnosis and management. Int Rev Neurobiol. 2008;83:259–71. doi: 10.1016/S0074-7742(08)00015-9 18929087

[5] Ma GJ, Yadav S, Kaplan PW, Johnson E. New-onset epilepsy in women with first time seizures during pregnancy. Seizure. 2020;80:42–5. doi: 10.1016/j.seizure.2020.05.022 32521501

[6] Trinka E, Cock H, Hesdorffer D, Rossetti AO, Scheffer IE, Shinnar S. A definition and classification of status epilepticus--Report of the ILAE Task Force on Classification of Status Epilepticus. Epilepsia. 2015;56(10):1515–23. 26336950 10.1111/epi.13121

[7] Rosenow F, Mann C. Status epilepticus in pregnancy. Epilepsy Behav. 2023;138:109034. doi: 10.1016/j.yebeh.2022.109034 36525922

[8] Harden CL, Hopp J, Ting TY, Pennell PB, French JA, Hauser WA, et al. Practice parameter update: management issues for women with epilepsy--focus on pregnancy (an evidence-based review): obstetrical complications and change in seizure frequency: report of the Quality Standards Subcommittee and Therapeutics and Technology Assessment Subcommittee of the American Academy of Neurology and American Epilepsy Society. Neurology. 2009;73(2):126–32. doi: 10.1212/WNL.0b013e3181a6b2f8 19398682 PMC3475195

[9] Battino D, Tomson T, Bonizzoni E, Craig J, Lindhout D, Sabers A, et al. Seizure control and treatment changes in pregnancy: observations from the EURAP epilepsy pregnancy registry. Epilepsia. 2013;54(9):1621–7. doi: 10.1111/epi.12302 23848605

[10] Beniczky S, Trinka E, Wirrell E, Abdulla F, Al Baradie R, Alonso Vanegas M, et al. Updated classification of epileptic seizures: Position paper of the International League Against Epilepsy. Epilepsia. 2025;66(6):1804–23. doi: 10.1111/epi.18338 40264351 PMC12169392

[11] Al-Mufti F, Claassen J. Neurocritical care: status epilepticus review. Crit Care Clin. 2014;30(4):751–64. doi: 10.1016/j.ccc.2014.06.006 25257739

[12] Norwitz ER, Hsu C-D, Repke JT. Acute complications of preeclampsia. Clin Obstet Gynecol. 2002;45(2):308–29. doi: 10.1097/00003081-200206000-00004 12048392

[13] Bilello LA, Greige T, Singleton JM, Burke RC, Edlow JA. Retrospective Review of Pregnant Patients Presenting for Evaluation of Acute Neurologic Complaints. Ann Emerg Med. 2021;77(2):210–20. doi: 10.1016/j.annemergmed.2020.02.014 32418678

[14] Pschirrer ER, Monga M. Seizure disorders in pregnancy. Obstet Gynecol Clin North Am. 2001;28(3):601–11, vii. doi: 10.1016/s0889-8545(05)70221-7 11512504

[15] Hauser WA, Annegers JF, Kurland LT. Incidence of epilepsy and unprovoked seizures in Rochester, Minnesota: 1935-1984. Epilepsia. 1993;34(3):453–68. doi: 10.1111/j.1528-1157.1993.tb02586.x 8504780

[16] Morton A. Imitators of preeclampsia: A review. Pregnancy Hypertens. 2016;6(1):1–9. doi: 10.1016/j.preghy.2016.02.001 26955764

[17] Man S-L, Petersen I, Thompson M, Nazareth I. Antiepileptic drugs during pregnancy in primary care: a UK population based study. PLoS One. 2012;7(12):e52339. doi: 10.1371/journal.pone.0052339 23272239 PMC3525559

[18] Katz VL, Farmer R, Kuller JA. Preeclampsia into eclampsia: toward a new paradigm. Am J Obstet Gynecol. 2000;182(6):1389–96. doi: 10.1067/mob.2000.106178 10871454

[19] Sibai BM. Diagnosis and management of gestational hypertension and preeclampsia. Obstet Gynecol. 2003;102(1):181–92. doi: 10.1016/s0029-7844(03)00475-7 12850627

[20] Thornton CE, Makris A, Ogle RF, Tooher JM, Hennessy A. Role of proteinuria in defining pre-eclampsia: clinical outcomes for women and babies. Clin Exp Pharmacol Physiol. 2010;37(4):466–70. doi: 10.1111/j.1440-1681.2009.05334.x 19930427

[21] Harsono AAH, Achmadi A, Aldika Akbar MI, Joewono HT. Recurrent Seizures in 2 Patients with Magnesium Sulfate-Treated Eclampsia at a Secondary Hospital. Am J Case Rep. 2018;19:1129–34. doi: 10.12659/AJCR.911004 30250014 PMC6180921

[22] Enye S, Ganapathy R, Braithwaite O. Proteinuria in status epilepticus or eclampsia; a diagnostic dilemma. Am J Emerg Med. 2009;27(5):625.e5-6. doi: 10.1016/j.ajem.2008.08.020 19497473

[23] Michalsen A, Henze T, Wagner D, Schillinger H, Engels K. Status epilepticus in der Spätschwangerschaft--Eklampsie oder Subarachnoidalblutung?. Anasthesiol Intensivmed Notfallmed Schmerzther. 1997;32(6):380–4. 9333337 10.1055/s-2007-995075

[24] Friedman HH. Problem oriented medical diagnosis. 7th ed. Philadelphia: Lippincott Williams and Wilkins; 2004.

[25] Allotey J, Fernandez-Felix BM, Zamora J, Moss N, Bagary M, Kelso A, et al. Predicting seizures in pregnant women with epilepsy: Development and external validation of a prognostic model. PLoS Med. 2019;16(5):e1002802. doi: 10.1371/journal.pmed.1002802 31083654 PMC6513048

[26] Lu Y-T, Hsu C-W, Tsai W-C, Cheng M-Y, Shih F-Y, Fu T-Y, et al. Status epilepticus associated with pregnancy: A cohort study. Epilepsy Behav. 2016;59:92–7. doi: 10.1016/j.yebeh.2016.03.034 27116537

[27] EURAP Study Group. Seizure control and treatment in pregnancy: observations from the EURAP epilepsy pregnancy registry. Neurology. 2006;66(3):354–60. doi: 10.1212/01.wnl.0000195888.51845.80 16382034

[28] Kusznir Vitturi B, Barreto Cabral F, Mella Cukiert C. Outcomes of pregnant women with refractory epilepsy. Seizure. 2019;69:251–7. doi: 10.1016/j.seizure.2019.05.009 31128468

[29] Rajiv KR, Radhakrishnan A. Status epilepticus in pregnancy - Can we frame a uniform treatment protocol?. Epilepsy Behav. 2019;101(Pt B):106376. doi: 10.1016/j.yebeh.2019.06.020 31303443

[30] Roberti R, Rocca M, Iannone LF, Gasparini S, Pascarella A, Neri S, et al. Status epilepticus in pregnancy: a literature review and a protocol proposal. Expert Rev Neurother. 2022;22(4):301–12. doi: 10.1080/14737175.2022.2057224 35317697

[31] Swor D, Juneja P, Constantine C, Mann C, Rosenow F, LaRoche S. Management of status epilepticus in pregnancy: a clinician survey. Neurol Res Pract. 2024;6(1):3. doi: 10.1186/s42466-023-00295-z 38233889 PMC10795404

[32] Razaz N, Igland J, Bjørk M-H, Joseph KS, Dreier JW, Gilhus NE, et al. Risk of Perinatal and Maternal Morbidity and Mortality Among Pregnant Women With Epilepsy. JAMA Neurol. 2024;81(9):985–95. doi: 10.1001/jamaneurol.2024.2375 39102246 PMC11385047

[33] Nordeng H, Ystrøm E, Einarson A. Perception of risk regarding the use of medications and other exposures during pregnancy. Eur J Clin Pharmacol. 2010;66(2):207–14. doi: 10.1007/s00228-009-0744-2 19841915

[34] Perucca P, Battino D, Bromley R, Chen L, Craig J, Hernandez-Diaz S, et al. Epilepsy-pregnancy registries: An update. Epilepsia. 2025;66(1):47–59. doi: 10.1111/epi.18180 39540312

[35] Li J, Toffa DH, Nguyen DK. Epilepsy and Pregnancy: An Audit of Specialized Care. Can J Neurol Sci. 2022;49(5):678–87. doi: 10.1017/cjn.2021.190 34353406

[36] Voinescu PE, Ehlert AN, Bay CP, Allien S, Pennell PB. Variations in Seizure Frequency During Pregnancy and Postpartum by Epilepsy Type. Neurology. 2022;98(8):e802–7. doi: 10.1212/WNL.0000000000013056 34893557 PMC8883510

[37] Mari L, Placidi F, Romigi A, Tombini M, Del Bianco C, Ulivi M, et al. Levetiracetam, lamotrigine and carbamazepine: which monotherapy during pregnancy?. Neurol Sci. 2022;43(3):1993–2001. doi: 10.1007/s10072-021-05542-2 34468899 PMC8860795

[38] Vajda F, O’Brien T, Graham J, Hitchcock A, Perucca P, Lander C, et al. Changes over 24 years in a pregnancy register - Teratogenicity and epileptic seizure control. Epilepsy Behav 2023; 148:109482. 37839246 10.1016/j.yebeh.2023.109482

